# Virtual Learning in Emergency Medicine Residency Programs

**DOI:** 10.1016/j.acepjo.2025.100069

**Published:** 2025-02-22

**Authors:** Ashley Rider, Laura Oh, Rahul Bhat, Michael Gottlieb, Bruce Lo, Ulemu Luhanga, Shayne Gue, Jason Laenngfeld, Sarah Greenberger, Jeffery Hill, Jonathan Heidt

**Affiliations:** 1 Department of Emergency Medicine, Stanford University, Palo Alto, California, USA; 2 Department of Emergency Medicine, Emory University School of Medicine Emory University, Atlanta, Georgia, USA; 3 Department of Emergency Medicine, Washington Hospital Center, Georgetown University, Washington, District of Columbia, USA; 4 Department of Emergency Medicine, Rush University Medical Center, Chicago, Illinois, USA; 5 Department of Emergency Medicine, Sentara Norfolk General Hospital/Eastern Virginia Medical School, Norfolk, Virginia, USA; 6 Department of Medicine, Emory University School of Medicine, Emory University, Atlanta, Georgia, USA; 7 Department of Emergency Medicine, University of Central Florida College of Medicine, Orlando, Florida, USA; 8 Department of Emergency Medicine, University of Nebraska Medical Center, Omaha, Nebraska, USA; 9 Department of Emergency Medicine, University of Arkansas for Medical Sciences, Little Rock, Arkansas, USA; 10 Department of Emergency Medicine, University of Cincinnati, Cincinnati, Ohio, USA; 11 Department of Emergency Medicine, School of Medicine, University of Missouri – Columbia, Columbia, Missouri, USA

**Keywords:** virtual learning, graduate medical education, emergency medicine

## Abstract

During the COVID-19 pandemic, educational systems worldwide faced significant disruptions as in-person learning became unfeasible. In response, many institutions, including graduate medical education programs, swiftly transitioned to virtual learning platforms to adapt to these challenges. The rapid and unplanned pivot in learning format resulted in temporary negative impacts on residency training. Conversely, the experience of the rapid shift may have resulted in some long-term benefits while also preparing programs for future disruptions. This review aimed to discuss the advantages and disadvantages of virtual learning, potential mitigation strategies for the realized disadvantages, and potential areas of future research.

## 1 Introduction

The COVID-19 pandemic caused massive disruption and forced evolution in the learning environment of emergency medicine (EM) residents, as traditional in-person EM didactics and conferences rapidly shifted to virtual platforms.^1^ This crisis provided an opportunity for educators to adapt to deliver core content that maintained the Accreditation Council for Graduate Medical Education standards while minimizing the risk of in-person contact. Once the restrictions on gathering due to the pandemic had been lifted, however, many programs reverted to familiar ways of teaching within the same physical spaces without incorporating technological advances and lessons learned during the pandemic. This represents a lost opportunity to fully realize the potential of virtual education as part of a hybrid curriculum or standalone curricular innovation. Reasons cited for a preference for traditional in-person didactics over virtual learning included concerns centered on learner engagement and lack of community.^2^

It is important to consider that virtual learning may have unrealized potential as it was widely introduced to graduate medical education in the context of an ongoing pandemic with unique psychosocial stressors. Due to the urgent need for rapid transition, virtual learning was implemented often without a stepwise process, grounding in educational theory, or modification to curricula based on resident feedback.^3^ Moving forward, with some strategic modifications targeted at highlighting the benefits of virtual conferences while addressing perceived limitations, virtual learning can play a role in future EM residency training.^4^ For this review, we have discussed virtual learning, with a focus on synchronous online learning.

## 2 Advantages of Virtual Learning

There are several advantages (Table) offered through virtual learning that may contribute to the overall educational experience for learners: access to national experts, the ability to recruit from a more diverse faculty speaker pool, increased collaboration with external institutions, increased efficiency of educational endeavors, flexibility in how information is presented and consumed, engagement of different kinds of learners, and the potential for improved well-being.

**Table:** Advantages and disadvantages of virtual learning.

Advantages	Disadvantages
• Collaboration with external institutions • Diverse speaker pool • No physical space requirements • Ease of recording • Flexibility in presentation and consumption of content • Chat engagement • Learner well-being	• Learner distraction or multitasking • Reduced social connection • Privacy concerns • Technology licensing cost • Technology failures • Limitations for procedural and hands-on learning • Instructor’s adaptation of content for a virtual platform

Virtual learning allows nationally and internationally recognized experts to present easily at external programs without the limitations of travel, time, and cost.^4^ Programs face fewer budget constraints, and emerging speakers may be more willing to forgo honoraria to gain visibility, enabling broader participation. During the pandemic, some programs engaged in shared didactics by pooling their faculty and learners.^5^ These shared learning environments provided students access to a larger faculty and relieved educators and institutions of the academic burden of teaching de novo virtual programming or content outside of their primary area of expertise.^5^

Virtual sessions also have the advantage of being more readily recorded for review at a later date. Recording reduces the need for faculty to repeat the same lecture and gives the learner more control over the speed of information consumption (eg, the ability to rewind, fast forward, and change listening playback speed). Virtual learning also offers the ability for more forms of engagement and increased capacity for participating in discussions. Virtual chat features offer learners the ability to ask clarifying questions in real time.^4^ This modality may also lower the threshold for engagement, potentially offering a safer option for introverts.^4^ Virtual conferences may also improve well being by repurposing commuter time for more rest, sleep, and self-care.^4^

## 3 Disadvantages of Virtual Learning

Perceived disadvantages (Table) of virtual learning include the potential for learner disengagement and distraction, reduced social connection, privacy concerns, technological limitations, and cost limitations with respect to procedural training and hands-on learning. Evidence is mixed on if virtual education is more distracting compared with in-person teaching and if there should be a “camera on” requirement.^4^^,^^6^ One multicenter study found that residents reported greater attention during virtual conferences compared with in-person didactics.^4^ Conversely, other recent studies of virtual didactics in EM, internal medicine, surgery, and anesthesiology suggest decreased engagement during virtual didactics.^7^, ^8^, ^9^, ^10^, ^11^, ^12^ One EM study of self-reported engagement suggests that participants engage in twice as many nondidactic-related activities when virtual.^7^

Virtual learning may also reduce social connections that foster well-being and community. Attendees surveyed about virtual conferences have reported decreased engagement with peers, less dialog among participants, and fewer opportunities to network compared with in-person conferences.^7^^,^^8^^,^^13^, ^14^, ^15^, ^16^ Seeing and interacting with colleagues can help build trusting connections between residents, faculties, and trainees, offer opportunities for informal mentoring, and support the well-being of the group.^4^ This loss of camaraderie may especially impact the well-being of new learners who lack local support systems. This may also have detrimental downstream effects for junior learners whose career development is enhanced by building relationships with career collaborators, mentors, and sponsors. Although there is limited data on the postpandemic influence of virtual didactics on burnout, 1 study of medical students suggested that online learning during the pandemic was associated with increased burnout compared with those who continued in-person learning.^17^

Privacy issues also create unique challenges in virtual learning. Peer review-protected activities, such as quality improvement or morbidity and mortality conferences, have increased privacy requirements. Care must be taken to ensure that online participants are not in public locations where content may be inadvertently shared.^18^

Barriers to successful virtual education implementation may also include technological barriers and cost. Network failure can render virtual learning impossible, whereas limited network capability can make the experience inconsistent.^19^ The cost of implementation and maintenance for software procurement and licensing, hardware and technology, and robust networks may hinder the adoption of best practices. Faculty members may not be aware of or be familiar with the various platforms available that might be used to enhance the virtual learning environment.

Challenges with procedural and hands-on experiences have also been expressed with virtual learning. The challenge of reproducing hands-on training creates a potential loss for learners in developing the manual dexterity for procedures.^20^ Virtual procedural training may also require more staff time to coordinate.^20^ Additionally, the acquisition and accessibility of equipment that can allow for reliable education may be an obstacle due to the logistical and financial cost of obtaining items such as mannequins or task trainers as well as medical equipment for the trainee. For example, virtual ultrasound training includes not only having equipment and software that is compatible with virtual education and readily available for the trainee but also having live models, which could include relatives, course participants, or patients, available to scan.^21^ Lastly, the inability of educators to teach without physically guiding the learning during a procedure can potentially decrease the effectiveness of teaching procedures.

## 4 Mitigation of Potential Disadvantages

The perceived disadvantages of virtual education can be addressed or mitigated by approaching the design of virtual education in a stepwise, organized fashion using a learning design framework.

Universal design for learning (UDL) is a framework, based on research into how humans learn, that is used to improve and optimize teaching and learning regardless of modality (virtual or in-person). UDL guidelines call for educators to design experiences that provide multiple means of engagement, representation, action, and expression.^22^^,^^23^

Multiple means of engagement can be achieved by providing options for recruiting interest, sustaining effort and persistence, and promoting self-regulation.^22^^,^^23^ Participants can act as cofacilitators and ideas and solutions can be crowdsourced by integrating tools such as chat, annotation, or whiteboard features. Other ways to maintain engagement during virtual didactics include adjusting the structure of didactics, eg, shortening sessions, incorporating more small-group breakouts, providing frequent breaks, and integrating polling and gamification into presentations.^4^^,^^6^^,^^7^

Multiple means of representation can be achieved by providing options for customizing the display of information, illustrating through multiple media, and highlighting patterns and relationships to support comprehension. Closed captioning features can be used in presentation software (eg, Google Slides, PowerPoint) or within the video conferencing platform (eg, Zoom and Teams). Online video (eg, YouTube) clips can be incorporated into presentations. Infographics can be utilized, in addition to annotation and whiteboard features within the platform. These features can also be integrated into various virtual teaching platforms such as Canvas, Moodle, Blackboard, and Google Classroom.

Multiple means of action and expression can be achieved by providing options for physical actions, expression and communication, and executive functioning.^22^^,^^23^ Built-in platform chat can be used in addition to reactions; polling software such as Poll Everywhere or Slido can be incorporated into presentations. Learners can share materials to supplement learning in real time, and can even engage in shared content cocreation to apply knowledge using GoogleDocs, Microsoft Word Online, or JamBoard (which will retire in December 2024).^4^ For more sensitive topics or brainstorms, Google products may allow for anonymity when commenting if users log out of their Google profile or use an incognito window to access the document.

Learning is reliant on social interactions through the social construction of knowledge and the development of robust communities of practice. Communities of practice are groups of individuals who unite around a common experience (eg, working in the Emergency Department), build relationships that allow them to share problems and solutions (eg, through case-based learning), and jointly build a shared set of resources and understanding (ie, the culture and practice of the group).^24^ Communities of practice can be built entirely within a virtual learning environment and mitigate feelings of isolation with trainees reporting that they feel more supported.^25^ Barnett describes a seven-step framework for the development of virtual communities of practice and found that trainees in their geographically isolated family medicine residencies felt supported and less isolated.^25^ This approach can be used in EM residencies, leveraging modern communication platforms (eg, Slack, GroupMe, and WhatsApp) to facilitate the sharing of ideas, problems, and solutions and develop a supportive virtual community of practice.

Educators need to approach future virtual didactics with an understanding that social interaction and communication are critical means of supporting both well-being and learning. It will be important to intentionally build opportunities for social interaction in virtual didactics, such as the use of break out rooms to host small group discussions, setting explicit camera-on expectations for small-group learning, and utilizing the chat function to facilitate synchronous online discussions that occur in parallel to presented didactics.^26^

## 5 Future Research

EM training embraced virtual learning at the start of the COVID-19 pandemic, but many programs reverted to in-person didactics in the aftermath.^5^ Questions remain as to what practices yield the most for learning and retention, as well as the optimal frequency of best-practice virtual instruction. Given the time, space, and personnel resources required for in-person education, the educational benefits should plausibly surpass the advantages of virtual education to justify its increased resource consumption. Currently, there is a lack of data on learner outcomes in virtual settings compared with in-person settings, and the studies are limited to Kirkpatrick model levels 1 and 2.^27^ Small studies suggest that virtual learning is noninferior to in-person learning; however, these have limited generalizability.^28^, ^29^, ^30^

Collective experience has demonstrated that repurposing an in-person lecture for a virtual setting and vice versa does not equate to an equivalent learner experience. Rather, educators should be encouraged to “design for online.”^31^ Future educational research should focus on specific topics or features of learning objectives that demand an in-person setting versus a virtual platform. For example, team-based learning (TBL) is a model for learning that allows for peer-based collaboration with the oversight of one facilitator.^32^ This pedagogy may be uniquely reformatted to adapt to the virtual setting with the appropriate educational content. In one comparative study, third-year medical students participated in a basic medical laboratory course as a flipped classroom (FC), in-person TBL, or online TBL in the setting of the COVID-19 pandemic.^33^ Learner performance on final examinations and experiment reports was similar in both TBL groups, but both outperformed the FC group. There was similar high learner satisfaction in the online versus the in-person TBL, and both groups were significantly higher than the FC approach.^33^ Subsequent studies should investigate the appropriate EM subject matter and learner context for not only TBL but also the efficacy of the online versus in-person methods of TBL delivery.

In a blended learning model of both virtual and in-person didactics, educators need an evidence-based approach to selecting which topics are fit for virtual learning and which are better suited for in-person. In order to determine which modality of learning is best fit for learning objectives, assessment tools of learner knowledge acquisition are necessary, but they currently have limited implementation in EM curricula beyond the annual In-Training Exam. Although this assessment may be helpful in preparing for the summative American Board of Emergency Medicine qualifying examination, it lacks detail that could guide educators in deciding which content should be taught virtually versus in-person. This is in contrast to the Undergraduate Medical Education medical curriculum that regularly implements assessment into blocks or topics of teaching and has shown success through online platforms in terms of both student satisfaction and learning outcomes.^34^ EM didactics should strive for standardized assessment practices across focused topic areas that allow for a direct comparison of virtual and in-person didactic efforts. This has more far-reaching implications, as it will hold programs to high standards of teaching the latest evidence-based practice as well as incorporate regular knowledge-based formative assessments.

If the social impact of in-person didactics is the rationale to continue, then the impact on well-being, engagement, and culture should be formally studied, particularly for a specialty with a high level of burnout.^35^ Real-time studies of relative social connectedness during virtual didactics versus in-person would be helpful to elucidate how in-person social interaction affects the resident trainee. In addition, focusing on the impact of educational content delivery on learners through well-being surveys or biannual meetings would be excellent sources of data. Future studies should focus on the impact of virtual didactic options on resource utilization, resident knowledge acquisition, and resident well-being to better understand how to optimize the inclusion of virtual learning.

## 6 Conclusion

There are clear benefits of virtual education with respect to increasing access to expert speakers, resource allocation, individualized learning opportunities, and resident well-being.^3^^,^^8^ Perceived disadvantages can be mitigated through the use of a stepwise, organized approach to virtual education based on learning theory and the creation of communities of practice that foster learner engagement. In the postpandemic era, further research is needed to determine how virtual learning formats may complement in-person learning environments to optimize EM didactics.

## Funding and Support

By *JACEP Open* policy, all authors are required to disclose any and all commercial, financial, and other relationships in any way related to the subject of this article as per ICMJE conflict of interest guidelines (see www.icmje.org). The authors have stated that no such relationships exist.

## Conflict of Interest

The article was written on behalf of the American College of Emergency Physicians Academic Affairs Committee.
